# Scrotal Abscess Drained by Iatrogenic Urethral Fistula in an Adult Diabetic Male

**DOI:** 10.1155/2017/9820245

**Published:** 2017-07-17

**Authors:** Marco Stizzo, Davide Arcaniolo, Carmelo Quattrone, Raffaele Balsamo, Marco Terribile, Celeste Manfredi, Vincenzo Mirone, Paolo Verze, Marco De Sio

**Affiliations:** ^1^Urology Unit, University of Campania “Luigi Vanvitelli”, Naples, Italy; ^2^Urology Unit, Department of Neurosciences, Sciences of Reproduction and Odontostomatology, University of Naples “Federico II”, Naples, Italy

## Abstract

A 46-year-old Caucasian male has been transferred to our urology department with a history of septic fever, uncompensated diabetes, pain, and scrotal swelling. On clinical examination, the left inguinal and scrotal area was swollen, tender, and painful; scrotal MR had been performed, showing the catheter tip in scrotal cavity and presence of gas. The case was diagnosed as scrotal abscess with urethroscrotal fistula. He was successfully treated with scrotal incision, drainage, catheter repositioning under fluoroscopic control, antibiotics, and insulin. This patient developed an infection of scrotum, which led to subcutaneous abscess getting worse by a poorly controlled glycemia. In this case, an iatrogenic fistula, caused by wrong catheterization, stops the evolving to a Fournier's Gangrene. Early detection and intervention provide opportunities to improve outcome of this disease.

## 1. Introduction

Fournier's Gangrene (FG) is characterized by a polymicrobial infection (aerobic and anaerobic bacteria) with an identifiable cause in 95% of cases, beginning in the genital or perineal region [1]; its mortality rate is high. We present the case of a patient with a penoscrotal abscess, an early stage of FG, drained by urethroscrotal fistula. In this case, the poorly controlled diabetes mellitus (DM) seems to be the main etiological factor. Urethroscrotal fistula is a rarely seen pathology in men [2]; it is frequently iatrogenic or secondary to urethral stones [3]. An iatrogenic urethroscrotal fistula, caused by an inappropriate indwelling catheter insertion, has been an unusual resolution.

## 2. Case Presentation

### 2.1. Patient's History

A 46-year-old Caucasian male has been transferred to our urology department in July 2016 from another hospital from our region. Relevant information about the patient's history included alcohol abuse, circumcision when he was child, acute hepatitis 5 years before observation, depression, and unknown DM on glycemic control. Patient reported an undocumented history of multiple stenosis of the anterior urethra, with persistent dysuria, treated for three times with internal urethrotomy by cold knife (Sachse's technique) in past years (last in May 2010). Sixteen days earlier, he had been admitted to internal medicine department complaining of septic fever, uncompensated glycemia, pain, scrotal swelling, and an acute urinary retention (AUR). A bladder catheter had been placed. A diagnosis of diabetes mellitus was established and it was complicated with penoscrotal abscess extending to surrounding subcutaneous tissue, with leukocytosis (24000 WBC/mL), C-reactive protein of 17 mg/dL, and serum creatinine of 2.1 mg/dL. He had undergone antibiotic therapy (imipenem 1000 mg TID IV and teicoplanin 400 mg BID IV) and insulin injection. After few days, the catheter had been removed, but the patient had undergone a new episode of AUR and the subsequent blind catheterization had been reported as “particularly difficult” in the discharge letter with no additional information. There was an improvement of fever, glycemia, and inflammatory markers after 3 days, despite the fact that a painful and swollen scrotal lump persisted.

**Figure 1:**
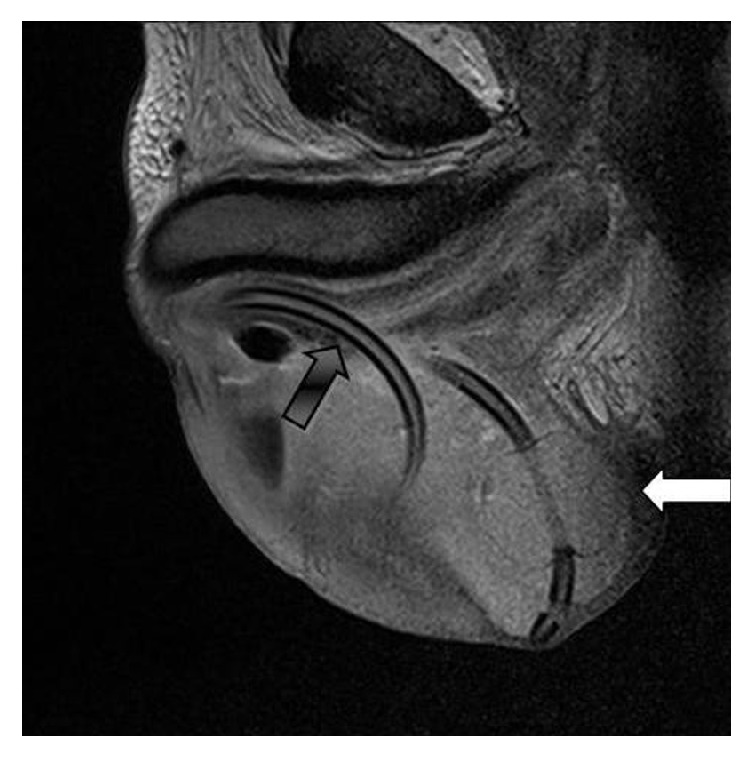
Scrotal MRI (sagittal): urethroscrotal fistula (black arrow) with catheter rolled in the scrotal sac, balloon (white arrow).

Before the patient was sent to our department, a scrotum MRI had been performed, showing the catheter tip in scrotal cavity and presence of gas (Figure 1).

### 2.2. Clinical Finding

On clinical examination, the left inguinal and scrotal area was swollen, tender, and painful; the skin was eroded and characterized by subcutaneous emphysema with crepitus. Scrotal abscess was diagnosed. Both testes and their epididymides did not show abnormal findings. Digital rectal examination showed a small, benign prostate, with mild pain at palpation. Supposedly an indwelling catheter was positioned in the bladder.

### 2.3. Diagnostic Assessment

Laboratory findings were as follows: white cell count of 6400/ml, hemoglobin of 10.4 g/dL, serum creatinine of 0.6 mg/dL, and blood glucose of 169 mg/dL.

### 2.4. Therapeutic Intervention

**Figure 2:**
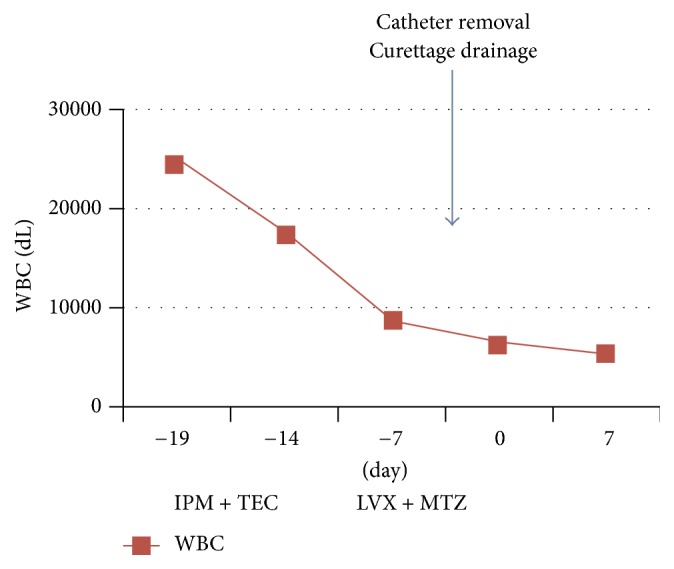
Clinical course. IPM, imipenem; TEC, teicoplanin; LVX, levofloxacin; MTZ, metronidazole.

Under fluoroscopic control, it was shown that contrast filled the scrotal sac. The old catheter was removed and a fistula from the bulbous urethra was shown. Finally a Mercier tip catheter 12 CH was inserted in the bladder. Retrograde cystography confirmed the correct positioning of catheter. Scrotal incision was performed and a Penrose drain was placed in the abscess. Approximately 200 ml of pus was evacuated from the abscess cavity. In order to prevent the risk of sepsis, we used metronidazole 500 mg QID IV and levofloxacin 750 mg QD PO (antibiotics sensibility was confirmed by a culture of a scrotal abscess). We washed the wound with physiological saline containing povidone iodine every day. As for the patient's diabetes mellitus, we carried out intensive control of blood glucose with subcutaneous injection of short- and long-acting insulin. After 7 days, there was a reduction of abscess on TC. The patient was discharged with oral antibiotics and catheter and reviewed weekly.  Figure 2 shows the white blood cells count during antibiotic therapy.

### 2.5. Follow-Up and Outcomes

**Figure 3:**
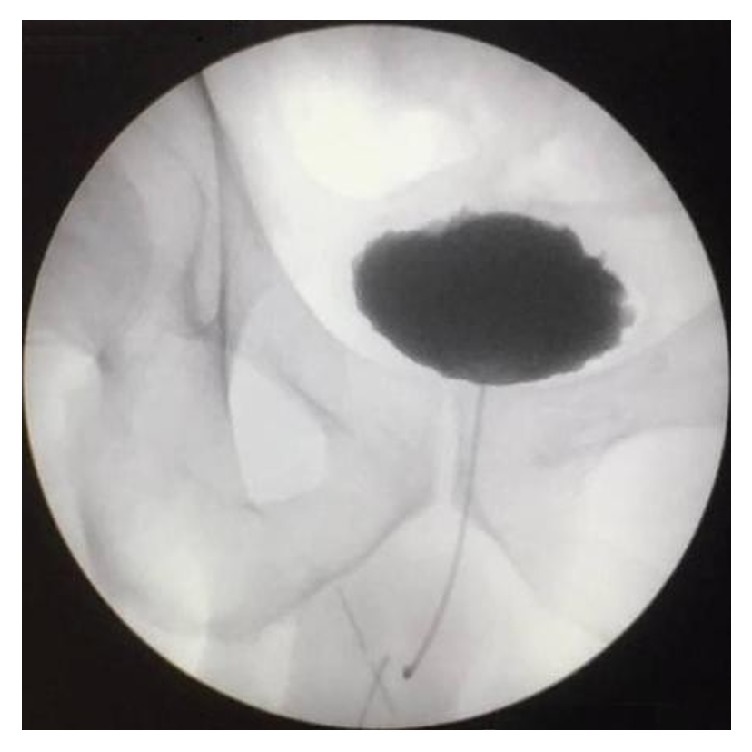
Retrograde cystography at 40th day.

**Figure 4:**
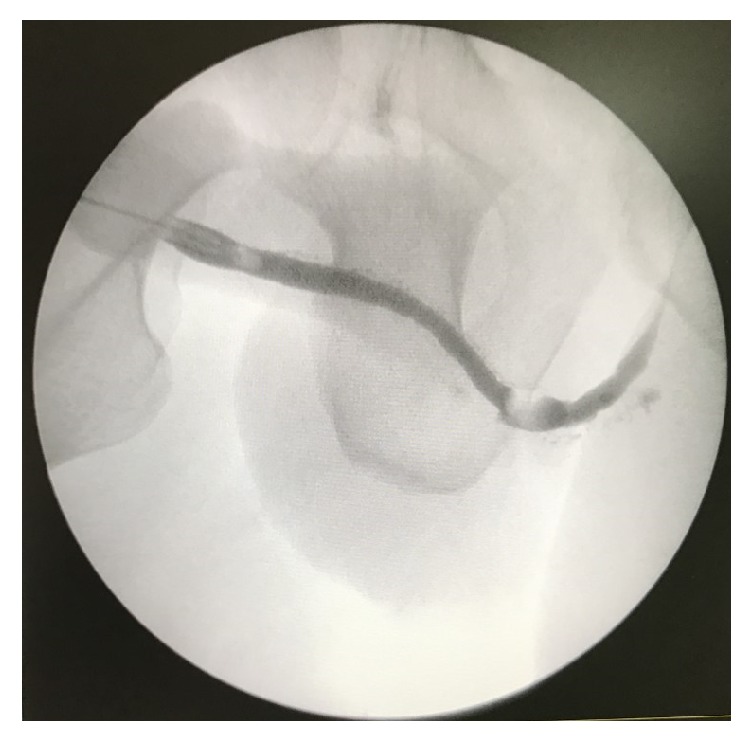
Retrograde urethrography at 10 months.

Since then the patient has presented regularly in our department. Drain was removed on the 20th day, a retrograde urethrogram was performed on the 40th day with no evidence of periurethral leakage (Figure 3), and the catheter was removed. He has not shown signs of recurrence in a follow-up period of 10 months (Figure 4).

## 3. Discussion

FG is an infection of male genitalia; rapid diagnosis and treatment are essential to reduce the morbidity and high mortality associated with this disease. There are well-documented associations between urethral obstructions, strictures, extravasation, and instrumentation. DM, trauma, periurethral extravasation, and surgery are usually predisposing factors [4]. This is a case of penoscrotal subcutaneous abscess in a young diabetic male, looking like FG. This patient developed an infection of scrotum, which led to subcutaneous abscess getting worse by poorly controlled DM. Luckily iatrogenic fistula, caused by wrong catheterization, drained the abscess and stopped the evolving of this case toward FG. Surgical debridement, glycemic control, antibiotic support, and maintenance renal function are the standard treatment for these cases in order to keep mean leukocyte count as low as possible. Leukocytosis was higher in patients who died than in surviving patients [5]. Adjunctive treatments such as hyperbaric oxygen and pooled immunoglobulin are of uncertain benefit and according to EAU guidelines are not recommended out of clinical trials [6]. We decided to not refer the patient to such therapies due to the absence of fever and the improvement of clinical symptoms.

In conclusion, it is important to evacuate abscess and pus as soon as possible and offer quick diagnosis and treatment in order to prevent the evolving of the disease.

Early detection and intervention provide opportunities to improve outcome of FG.
